# Neuroinflammation and Prefrontal Cortical Microcircuits: A Narrative Review of Hints from Experimental Models

**DOI:** 10.3390/cells15100869

**Published:** 2026-05-09

**Authors:** Maria Concetta Geloso, Gabriele Di Sante, Alberto Granato

**Affiliations:** 1 Section of Human Anatomy, Department of Neuroscience, Università Cattolica del Sacro Cuore, Largo Francesco Vito 1, 00168 Rome, Italy; 2 Gemelli Science and Technology Park (GSTeP)-Organoids Research Core Facility, Fondazione Policlinico Agostino Gemelli IRCCS, 00168 Rome, Italy; 3 Section of Human, Clinical and Forensic Anatomy, Department of Medicine and Surgery, University of Perugia, P.le L. Severi 1, 06127 Perugia, Italy; gabriele.disante@unipg.it; 4 Department of Veterinary Sciences, University of Turin, Grugliasco, 10095 Turin, Italy

**Keywords:** neuroinflammation, prefrontal cortex, cortical microcircuit, pyramidal neurons, interneurons, excitation/inhibition balance, somatostatin interneurons, vasoactive intestinal peptide interneurons, parvalbumin interneurons

## Abstract

Neuroinflammation is increasingly recognized as a major contributor to cognitive and neuropsychiatric dysfunction. The prefrontal cortex (PFC), a key substrate for executive functions and emotional behaviors, appears particularly vulnerable to inflammatory insults due to its high metabolic demand, prolonged developmental trajectory, and specialized microcircuit organization. In this narrative review, we focus on evidence from experimental models of neuroinflammation affecting the PFC. In particular, we approach the topic from the novel perspective of how neuroinflammation affects PFC microcircuitry, exploiting evidence from experimental models. The impact of neuroinflammatory processes on PFC microcircuit players, namely pyramidal neurons and different classes of inhibitory interneurons, will be examined and discussed. We also examine regional features that may underlie PFC susceptibility. Although available findings support the idea that neuroinflammation destabilizes PFC microcircuits, marked heterogeneity across models, timing, inflammatory burden, and readouts still limits direct comparison across studies. A more mechanistic and longitudinal understanding of these changes will be essential to clarify how inflammatory insults reshape PFC computation and contribute to cognitive dysfunction.

## 1. Introduction

Neuroinflammation refers to a spectrum of inflammatory responses within the central nervous system (CNS), involving resident glial cells, vascular elements, and, in some conditions, infiltrating peripheral immune cells. Rather than representing a single disease entity, neuroinflammation is a pathophysiological process that can arise in response to pathogen-associated molecular patterns, damage-associated molecular patterns, autoimmunity, tissue injury, or systemic inflammatory signals [1,2,3]. All these triggers activate microglia, the resident immune cells of the CNS. In the healthy adult brain, microglia continuously survey the local microenvironment, playing a crucial role in maintaining homeostasis, synaptic remodeling and immune surveillance [3,4]. Following inflammatory stimulation, however, microglia can adopt reactive phenotypes characterized by phagocytic activity, chemotactic properties and amoeboid morphology (for review see [5]). They can also develop a non-phagocytic phenotype, characterized by cytokine release (for review see [5,6]), interactions with astrocytes [7] and vascular cells, thereby amplifying local inflammatory signaling [8,9,10]. Astrocytes, in turn, can either support tissue homeostasis or contribute to maladaptive inflammatory responses. For instance, molecular signals transmitted through the syncytium in which astrocytes are organized might contribute to the recruitment of additional astrocytes [9]. Furthermore, astrocytes can recruit distant microglia in the brain parenchyma [10], leading to further involvement of other astrocytes, thus enhancing the inflammatory signal [11].

Proinflammatory mediators released by resident or infiltrating immune cells play a critical role in both the structural and functional alterations of neurons. It is established that neuroinflammation affects neuronal integrity through various mechanisms [12]. Due to the selective vulnerability of synapses [13], the impairment of the synaptic compartment is emerging as one of the earliest events associated with neuronal damage across a broad range of neurological diseases, as it is evident from both clinical and preclinical evidence [13,14,15,16]. Principal pathogenic events include aberrant microglia-mediated synaptic pruning [17], synaptic stripping [12,18], and alterations in the expression patterns of pre- and post-synaptic proteins, which likely reflect functional impairment of synapses [19]. Neuronal structural damage mediated by neuroinflammation also involves axonal degeneration [12,20]. Key pathogenic events in inflammation-mediated axonal degeneration include activation of inflammatory pathways, such as caspase-6 [20], oxidative stress [21], and axonal transport failure [22]. Furthermore, the process is exacerbated by the disruption of axonal transport of mitochondria, leading to reduced energy production and, consequently, to neurodegeneration [23]. Changes in the expression and distribution pattern of several ion channels, including calcium [24] and sodium channels [25], have been implicated in the pathophysiology of neuronal damage under inflammatory conditions, thereby altering neuronal functional properties [26]. Therefore, beyond structural damage, neuroinflammation can affect neuronal functions and neural plasticity, including long-term potentiation (for review see [6]), and excitatory/inhibitory (E/I) balance [27]. Together, these findings suggest that neuroinflammation contributes to CNS functional impairment by affecting neuronal function.

On this basis, a growing body of evidence supports the connection between neuroinflammation and impairment of neurological functions, including cognitive abilities, particularly when inflammatory signals impact brain regions involved in executive functions and emotional regulation [1,11,28]. Among these, the prefrontal cortex (PFC) is of special interest because it supports working memory, behavioral flexibility, attention, and top-down control, functions that are particularly vulnerable to inflammatory disturbances [29].

However, the literature remains fragmented across disease-specific frameworks and exhibits highly heterogeneous inflammatory paradigms.

In this regard, the present review synthesizes how inflammatory processes affect PFC structure and function. Rather than providing a broad overview of all inflammation-associated neurological and psychiatric disorders, we examine how inflammatory insults alter the PFC functional unit, i.e., the cortical microcircuit. This novel perspective highlights alterations in microstructure, morphology, and intrinsic local functions that could be relevant for the understanding of changes that contribute to cognitive and behavioral disturbances consequent to inflammatory conditions.

## 2. Overview of Anatomic and Functional Features of PFC

### 2.1. Definition of PFC

The PFC represents the highest level of the cortical hierarchy and is considered the anatomical substrate of complex mental processes and abstract functions, such as selective attention, abstract reasoning, complex learning, inhibition of impulses, and even consciousness [30,31,32].

Topographically, the PFC corresponds to the anterior part of the frontal lobe, extending anterior to the motor cortices. However, the lack of clear boundaries makes this definition imprecise [33]. Since a distinct layer 4 (L4) separates “frontal” (agranular) from “prefrontal” (granular) regions of the frontal lobe, Brodmann based the definition of this region on cytoarchitectonic criteria [30,33]. However, this interpretation was controversial because it seemed not to include rodents, which, on one side, lack L4 in the frontal areas, but, on the other hand, are the most common animal models used in neuroscience research [30,34,35]. The current definition of PFC relies on Rose and Woolsey’s old proposal to use the projection field of the mediodorsal (MD) thalamic nucleus, which targets a specific area of the frontal region in both primates and non-primate mammals (reviewed in [29,30,36]), including rodents [31]. However, since other thalamic nuclei also project to the PFC, this criterion is not sufficient on its own [30]. Therefore, due to the significant homology across mammalian species, PFC-basal ganglia-thalamo-cortical circuits are also considered useful to improve the definition of PFC, making it more inclusive [30].

According to functional and connectivity features, the PFC includes two main regions: (i) the orbitofrontal (OFC) and ventromedial PFC (vmPFC); (ii) the lateral [dorsolateral (DLPFC) and ventrolateral (VLPFC)] and dorsomedial PFC (DMPFC) [33]. Orbital and ventromedial subdivisions can be further subdivided into the infralimbic (IL), the prelimbic (PL), and the anterior cingulate cortices (ACC) [34]. They are functionally distinct, since OFC and vmPFC mature earlier and are involved in the control of emotional behavior [37], while DLPFC represents the last in maturation and is concerned with high cognitive functions [30,33,37]. Recently, using large-scale neuronal recordings, it has been proposed that maps based on the firing pattern provide a further criterion for the subdivision of PFC [38].

### 2.2. Principal Connections of PFC

The broad range of functions performed by the PFC is strictly dependent on its connections. Among them, thalamic projections are particularly relevant, since it is widely described that PFC activity depends on its thalamic counterpart [39]. The most important connections are those with MD, which are organized in topographically ordered parallel loops [39]. Since patients with MD lesions show deficits in executive functions similar to those observed in individuals with frontal lobe dysfunction, PFC-MD circuits are widely considered the anatomical substrate of PFC’s main functions, such as working memory, goal-directed and flexible behaviors, and reversal learning [39,40]. Notably, these reciprocal connections are altered in many neurologic and psychiatric diseases associated with emotional and cognitive impairment, including schizophrenia, bipolar disorders [41], Alzheimer’s Disease (AD), and frontotemporal dementia [42].

The PFC is also extensively connected with other cortical areas that differ across its various subdivisions. The lateral PFC receives inputs from (unimodal) association sensory cortices, such as visual areas, which target area 8 and are involved in top-down modulation of visual processing [43], or auditory areas, which target area 10 and are essential for higher-order auditory processing (for review see [44]).

Conversely, the OFC receives both sensory inputs, including those coming from primary sensory cortices, such as the primary olfactory cortex, and inputs from heteromodal association areas, such as the entorhinal cortex, thus collecting information from both the external and internal environment [44]. Highly specialized and strong reciprocal connections of the posterior portions of both medial PFC and OFC with the amygdala, which receives multiple sensory information from several association cortices, contribute to the integration of information from external and internal environments, related to emotionally relevant events [44,45]. These pathways are evolutionarily conserved and contribute to ongoing cognitive processes such as learning and memory, as well as to many aspects of social behavior, including social decision-making [46]. Reciprocal basolateral amygdala–PFC-mediodorsal thalamic connectivity is critical for assigning emotional salience to sensory and contextual information [47].

The PFC is also tightly connected with motor cortical areas, such as the premotor cortices and the supplementary motor area, and with non-cortical regions involved in motor functions, such as the cerebellum and the basal ganglia [48,49,50]. Prefrontal-striatal and cerebellar loops contribute to behavioral selection, action updating, and adaptive control [47]. In particular, the DLPFC projects to the rostral caudate nucleus [51] and related pallidal efferents are mediated by motor thalamic nuclei [44,51]. The OFC projects to the ventral striatum, which is functionally associated with the limbic system, and, together with the medial PFC, controls visceromotor centers, such as the hypothalamus, the solitary nucleus, and the rostral ventrolateral medulla [44]. In this way, the PFC controls autonomic and endocrine responses to emotions [52] and stress responses [53].

Framing these connections functionally is important because neuroinflammatory disruption of any of these pathways may translate into distinct cognitive, emotional, or motivational deficits.

### 2.3. Summary of Specific Features of PFC Microcircuits

#### 2.3.1. Cortical Microcircuits: Computational Units of the Neocortex

After the seminal discoveries of Vernon Mountcastle, the neocortex has been viewed as an array of radially oriented modules, usually referred to as columns (see [54], for review). Thereafter, the concept of cortical columnar organization has been variously interpreted and subtle, often confusing differences among columns, minicolumns, and microcolumns have been introduced (reviewed in [55,56]). However, even considering tangential intercolumn long- and short-range communication, any description of cortical microcircuits is still based on the assumption that the geometry of cortical computation is columnar. With this proviso, we focus here on a few basic aspects of cortical microcircuitry. First, the “canonical” circuit relies on the radial spread of intracortical excitation, initially generated by a volley of thalamic input. Spiny stellate, excitatory cells, of the thalamo-recipient L4 project to pyramidal neurons (PNs) of L2/3 (supragranular layers), and they in turn synapse onto infragranular pyramidal neurons of L5, which provide the output from the cerebral cortex [57]. This oversimplified, vertically oriented flow of synaptic signals is far from describing the complex diagram of connections among excitatory neurons. Glutamatergic, excitatory PNs account for about 80–90% of neocortical cells. In addition to modified PNs of L4 and L2/3-L5 PNs, a multitude of PNs reside in L6, provide feedback projections to the thalamus, and display distinctive anatomo-functional features [58,59]. Moreover, despite sharing a similar morphology, characterized by the subdivision of their huge dendritic tree into basal and apical domains, PNs are not a homogeneous population. In fact, they feature interareal [60,61], as well as interlaminar (i.e., L5 vs. L2/3) differences (e.g., [62]). Basal and apical dendrites of PNs represent distinct, though interacting, compartments: basal dendrites receive bottom-up input from the thalamus or areas that are located at lower levels of the cortical hierarchy, while the apical dendrite is the recipient of top-down, feedback connections from higher cortical areas or from thalamic nonspecific nuclei [63,64,65]. The interaction between apical and basal dendrites ensures the integration between inputs coming from the external world and context-related top-down information [64,66,67]. The apical-basal interplay is thought to play a crucial role in cognitive functions [68] and might be decisive in bridging the gap between cortical physiology and deep neural networks [69].

**Figure 1:**
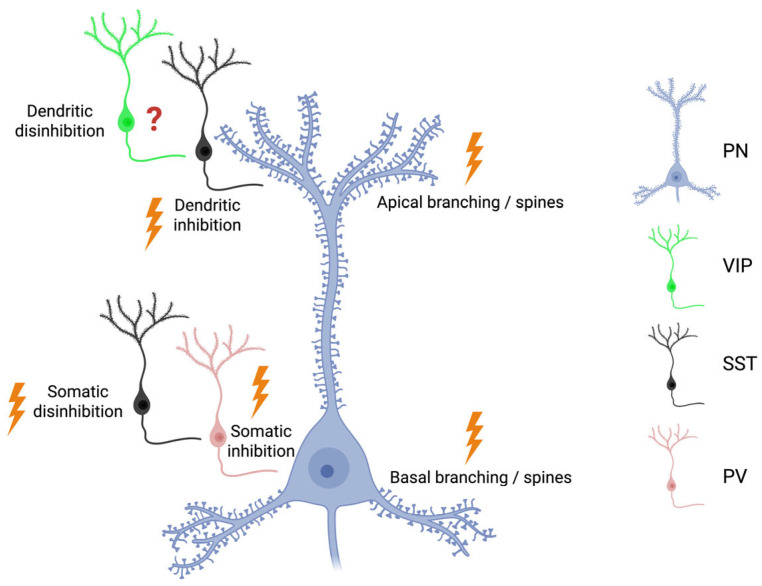
Summary diagram showing the circuit linking PNs and INs in PFC. Inflammatory processes (lightning symbol) can affect basal and apical dendrites of PNs, dendritic inhibition mediated by SST INs, and somatic inhibition/disinhibition mediated by the SST-PV circuit. Evidence for dendritic disinhibition mediated by VIP interneurons in this context remains limited (question mark). IN: Interneuron; PV: parvalbumin; SST: somatostatin; VIP: Vasoactive intestinal peptide; PN: Pyramidal neuron. Created in BioRender. Di Sante, G. (2026) https://BioRender.com/6w7nmca (accessed on 5 May 2026).

Inhibitory GABAergic interneurons (INs) represent the remaining cortical neurons (about 10–20%). Their morphological and functional features are manifold [70]. However, for the sake of simplicity, they can be divided into three non-overlapping populations, with different involvement in cortical microcircuits: parvalbumin (PV) fast spiking INs target the body and axon initial segments of PNs, whereas somatostatin (SST) INs synapse onto dendrites of PNs. Finally, vasoactive intestinal peptide (VIP) INs, a subpopulation of serotonin receptor 3a (5HT3aR) INs, contact SST INs, thus mediating disinhibition of PNs [71]. Interestingly, in recent years, it has become clear that neocortical INs engage in complex interconnections that modulate several aspects of PNs function, with special regard for the above-cited top-down projections to the apical dendrite (Figure 1). Long-range excitatory feedback projections from higher cortical areas contact VIP INs, which in turn inhibit SST INs, with disinhibition of the apical dendrite of PNs as a final effect ([72]; see also Figure 2 in [73]). Therefore, owing to the relevance of the apical mechanisms for cognition (see above), this disinhibitory circuit is thought to be critically involved in functions such as selective attention [74] and contextual modulation [75,76].

#### 2.3.2. Specific Features of the PFC Microcircuits

Specific features in cell morphology and density of key elements of the cortical microcircuit represent the structural basis of functional properties of the PFC. It is known that PNs display an increasing level of morphological complexity moving from areas involved in early stages of information processing, such as primary sensory regions, to those engaged in more complex activities [77]. They also exhibit significant structural differences among primate species, showing a larger soma, increasingly complex dendritic trees, and increased numbers of spines in species in which the granular PFC covers a larger cortical area [78]. In monkeys, the mean somato-pia distance for DLPFC L3 PNs is significantly greater than in the primary visual cortex, indicating their larger size. Moreover, they show an increased number of dendritic branches, which correlates with the level of information processing complexity across different cortical areas [60,61,79,80,81]. This is also true for PFC L5 PNs, which exhibit increased dendritic complexity, particularly in basal dendrites, compared to those in sensory areas [79], as well as a distinctive morphology of the apical dendrite [82]. The latter led to the subdivision of L5 PFC PNs into different subpopulations that differ in their response to synaptic stimulation [82,83,84]. The apical dendrite also shows particular morphological features, based on which PNs are subdivided into thick-tufted, type A neurons, that project subcortically with prominent hyperpolarization-activated current (Ih), and thin-tufted, type B neurons, which generate callosal projections and lack Ih current [85]. They receive distinct input, different IN inhibition, which results in different functional properties [86].

From a functional point of view, the key feature of PFC PNs is their ability to fire persistently in response to transient stimuli, during the delay period of working memory tasks [80,87]. In addition to the recurrent network activity, a few intrinsic properties of PFC PNs, including the modulation of Hyperpolarization-activated Cyclic Nucleotide-gated (HCN) channels and the postburst afterdepolarization (sADP), may contribute to the generation of persistent activity [88]. HCN channels, responsible for the Ih current, are gated by cyclic nucleotides [89]. Notably, HCN channels modulate the interaction between the information processing in apical and basal dendrites [90]. Their density in distal branches of the apical dendrite is increased during development, thereby favoring the transition of PNs from compact single-point to two-point (basal and apical) integrators [91,92]. Furthermore, the Ih current is modulated by the ascending noradrenergic cortical input [90]. Persistent activity of PFC PNs is induced by the downregulation of HCN channels, mediated by adrenergic modulation [93] or by the activation of metabotropic glutamate receptors [88]. The negative modulation of the Ih current can also be responsible for an increased sADP [88]. Furthermore, the adrenergic activation can modulate dendritic calcium spikes generated by backpropagating action potentials through the inhibition of the Ih current [94]. Different from PNs of the somatosensory cortex, HCN channels are mainly located in dendritic spines, colocalized with a constellation of synaptic- and modulatory-related molecules and conductances [93,95,96]. As mentioned before, PFC PNs are not uniform from the electrophysiological point of view, as distinct subthreshold properties and distributions of dendritic ion channels depend on whether they project to telencephalic or to subcortical structures [88,97,98].

The sADP, which is critically involved in sustaining PNs firing during the delay period, is usually determined by the Ca^2+^ electrogenesis at distal apical dendrites [99]. However, dendritic Ca^2+^ spikes in some classes of PFC PNs may differ from those observed in other neocortical areas. A role of voltage-gated Ca^2+^ channels (VGCCs) for the generation of sADP in PFC neurons has been shown by [100]. These data are in good agreement with the findings of dendritic VGCCs and Ca^2+^ spikes in PL PNs [101]. It has been found that T-type VGCCs display higher expression in PFC neurons, as compared to other cortical areas [102]. Conversely, in the ACC, L5 PNs do not display prominent dendritic Ca^2+^ spikes, even though, because of the compact geometry of their apical dendrites, they are still able to propagate efficiently slow signals, such as N-methyl-D-aspartate (NMDA) spikes [103]. The role of NMDA signaling in sustaining the persistent firing is also suggested by the strong reliance of PFC neurons on NMDA neurotransmission [104,105]. The role of Ca^2+^ influx through NMDA and VGCCs for the generation of sustained firing is reviewed in [106].

The geometric and topological properties of the dendritic tree represent key determinants of neuronal firing patterns (e.g., [79,107,108]). Therefore, some of the electrophysiological features of PFC neurons can be attributed to their particular pattern of dendritic branching (see [82]).

The role of dopamine in modulating the functional properties of PFC neurons is well established (see [109], for review), and its influence on dendritic Ca^2+^ spikes and sADP might depend on the type of dopamine receptor activation. Yang and Seamans [110] report a downregulation of dendritic spikes following D1 stimulation, while their enhancement has been demonstrated after D2 agonist application [100].

In addition to PNs, the PFC is characterized by a specific arrangement and density of diverse IN subtypes, whose differences in cell-density, spatial distribution, and connectivity contribute to the regional specialization of the cerebral cortex [60,61,111,112]. In the PFC, PV+ cell density is lower, and the SST+ cell density is higher than in sensory areas [111,112]. Specifically, this rostral-caudal gradient pertains to the subpopulation of SST+ INs co-expressing calretinin, while SST/nNOS+ cells exhibit a more uniform distribution across the neocortex, similar to the VIP subgroup of interneurons [111]. The latter, in mPFC, inhibit SST+ INs and, to a lesser extent, PV+ INs, to promote PNs’ activity [76]. Moreover, increased numbers of cholecystokinin (CCK)+ INs, a subset of 5HT3aR INs that exert perisomatic inhibition [113,114], characterize the rodent mPFC [115,116]. The PFC also contains specific subtypes of INs, such as choline acetyltransferase (ChAT)-VIP neurons, which directly excite neighboring neurons across cortical layers, thereby contributing to attention [117]. Since INs shape the response of PNs to both local and long-range excitatory inputs, their specialized arrangement in the PFC significantly influences the functional properties of this cortical region.

## 3. Susceptibility of PFC to Neuroinflammation

The susceptibility of the neocortex, especially the PFC, to neuroinflammation is supported by several studies [29,118,119], and recent evidence in rodents points to the presence of regionally specific inflammatory profiles among different areas of the cerebral cortex [120].

**Figure 2:**
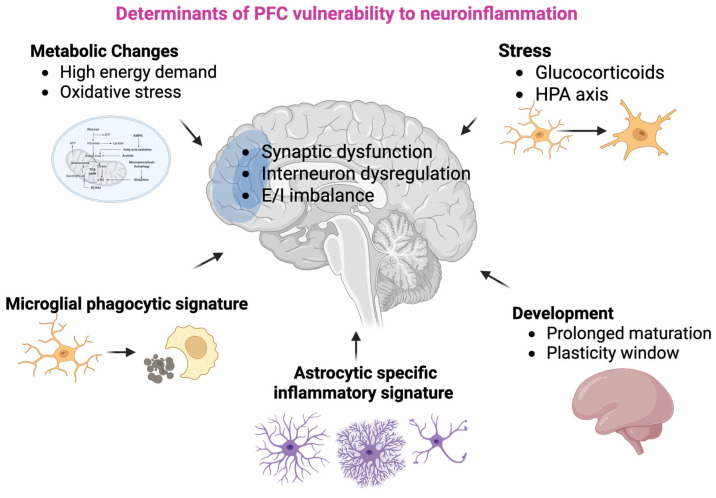
Schematic representation of different factors playing a role in prefrontal cortex (PFC) susceptibility to neuroinflammation. This figure summarizes the main factors contributing to the selective vulnerability of the PFC to neuroinflammatory insults. High metabolic demand, prolonged developmental maturation, glial specialization, and stress-related signaling converge to affect PFC microcircuits, ultimately leading to synaptic dysfunction and excitation/inhibition imbalance. Created in BioRender. Di Sante, G. (2026) https://BioRender.com/6afea31 (accessed on 5 May 2026).

A distinctive transcriptional profile of PFC has also been shown in the Rhesus macaque monkey during SHIV1 viral infection, characterized by enrichment of genes regulating T cell differentiation and microglial activation [121]. This is in line with the knowledge that, across the cortical hierarchy, there are significant differences in neurotransmission, neuromodulation, and signaling pathways (e.g., the calcium pathway), which make neurons in the association cortices more vulnerable to noxae than those in sensory cortices, such as the visual cortex (for review, see [106]). Consistent with this, although limited to the mouse model of intracisternal lipopolysaccharide (LPS) administration, there is evidence indicating that, upon inflammatory challenge, the murine PFC exhibits the highest level of activated microglia compared to other cortical or subcortical brain regions [29]. In addition, with the limitations of an in vitro study, recent data suggest that this area also exhibits increased susceptibility to tumor necrosis factor (TNF)-α-induced inflammation compared to other brain regions [122]. Overall, these findings support the hypothesis that the PFC could be particularly vulnerable to inflammatory insults. Although the neurobiology of this phenomenon remains unclear, some hypotheses have been proposed (Figure 2).

Structural features of the PFC (see Section 2) can affect its susceptibility to neuroinflammation. For instance, the increased complexity of the dendritic tree that characterizes its PNs is associated with high metabolic requirements [123]. Moreover, the PFC shows a higher metabolic rate compared with other brain regions [124], which makes this cortical area particularly susceptible to the disruption of energy metabolism induced by acute inflammation (Figure 2). It is known, in fact, that neuroinflammation induces mitochondrial dysfunction (for review see [125]) and hypoglycemia [126], leading to increased reactive oxygen species (ROS) production and altered calcium buffering, with consequent impairment of neuronal activity.

Neuroinflammation is also linked to several other changes in cellular metabolism, such as disruption of aspartic acid metabolism, upregulation of the urea cycle [127] and/or alterations of tryptophan metabolism [128]. In this regard, some authors have hypothesized that kynurenic acid, a byproduct of tryptophan catabolism that increases during neuroinflammation [129], might contribute to the vulnerability exhibited by the PFC [130,131] (Figure 2). Kynurenic acid acts as an antagonist on NMDA receptors, which play a fundamental role in generating persistent firing in the absence of sensory stimulation that characterizes DLPFC neurons, representing the basis of working memory and mental representation [132]. Notably, recent studies on primates have shown that the antagonistic effect on NMDA and nicotinic α7 receptors of L3 PFC PNs markedly reduces the delay-related firing needed for working memory [133]. Kynurenic acid derives from kynurenine by KATII, encoded by the gene AADAT. Interestingly, the same group highlighted a large expansion of KAT II/Kynurenic acid signaling in the primate DLPFC, compared to rodents [133], thus highlighting the specificity of this pathway in the context of PFC function and response to inflammation.

Another specific feature of PFC is its prolonged myelination [134]. Since a specific relation between gut microbiota and PFC myelination has been discovered [135], and considering that dysbiosis is believed to contribute to the pathogenesis of neuroinflammation [136], we may speculate that this could be one of the factors contributing to PFC susceptibility to inflammatory processes.

Moreover, as extensively reviewed by others, the PFC exhibits a particular sensitivity to stress (see [130,137,138]), which induces neuroendocrine dysfunction, increased catecholamine release, changes in structural plasticity, and low-grade inflammation [137,138]. Although the revision of the consequences of stress-induced low-grade inflammation goes beyond the aims of the present review (see Section 6 for inclusion criteria), it should be noted that the predisposition of PFC to stress, in turn, increases its vulnerability to inflammation by promoting microglial activation and subsequent release of proinflammatory molecules [139]. Indeed, high levels of inflammatory mediators released during chronic stress prime microglia [140], thereby enhancing their capacity to respond to inflammatory stimuli. Moreover, due to its prolonged period of postnatal development, the PFC is particularly susceptible to a variety of early-life stressors [137]. In this regard, Dinel and colleagues highlighted that neonatal inflammation increases the PFC’s susceptibility to inflammatory events in adulthood [141] (Figure 2).

The presence of proper characteristics of microglia and astrocytes may provide a significant contribution to PFC-specific changes and vulnerability to neuroinflammation. For instance, it has been shown that the transcriptional signature of murine PFC microglia differs from that of other brain regions, such as midbrain and striatum, and is particularly enriched for synapse-related pathways [142]. This feature seems specifically oriented to refine local circuits through synaptic pruning, especially during development and may reflect the specific functions of PFC [143]. This activity may turn into aberrant synaptic pruning in some pathological conditions associated with neuroinflammation, such as Multiple Sclerosis [15], schizophrenia [144], depression [145], and alcohol abuse [146] (Figure 2).

Consistently, there are findings highlighting the regional specificity of astrocyte phenotypes and functions in different brain regions [147,148], including the cerebral cortex [149]. In this regard, Diaz-Castro and colleagues, through astrocyte-specific RNA-sequencing experiments, demonstrated that PFC astrocytes show a proper regional transcriptional signature that is different, for instance, from that of other cortical areas, such as the visual cortex [149]. In addition, from a functional point of view, PFC astrocytes are activated by different spatiotemporal patterns of intracellular Ca^2+^ and by specific complex stimuli compared to those in sensory areas [150]. Combining in vivo Ca^2+^ imaging with genetic silencing of astrocytes, Kim and colleagues identified a specific role of astrocyte Ca^2+^ signaling within the mPFC in anxiety regulation [151]. In addition, they also demonstrated a possible role of mPFC astrocytes in maintaining E/I balance, since suppression of their activity induced increased glutamatergic transmission and decreased GABAergic transmission in the mPFC [148]. It is known, in this regard, that dopamine transmission regulates many temporally and functionally different processes in the PFC, including attention, working memory, behavioral flexibility, action planning and affective states [152]. Together, these findings strongly suggest that, due to their specific features, PFC astrocytes may play a key role in modulating inflammatory responses and neurovascular coupling, and emerging evidence suggests that region-specific astrocytic phenotypes may contribute to differential vulnerability of the PFC. They may also play a region-specific role in the context of neuroinflammation, as pointed out by Diaz-Castro and coworkers, who found that, upon peripheral challenge with LPS, PFC astrocytes show a specific inflammatory signature, that, in addition to activation of immune signaling, includes pathways involved in homeostatic roles such as cholesterol metabolism, cytoskeleton dynamics, gap junctions, K^+^ and Ca^2+^ transport, synapse plasticity, and signaling to other cells. No evidence of neuronal damage emerges in this experimental context, suggesting that, in the PFC, astrocyte reactivity is not associated with a decrease of its homeostatic role in neural functions [149] (Figure 2).

Thus, PFC vulnerability should be viewed as a multidimensional property emerging from the interaction among metabolic load, developmental timing, glial specialization, and circuit architecture, rather than as the consequence of a single inflammatory mechanism.

## 4. Effects of Neuroinflammation on Neuronal Elements of the PFC Microcircuit

### 4.1. Pyramidal Neurons and Neuroinflammation Microcircuits

Referred to as “the psychic cell” by Ramon y Cajal, PNs not only represent the majority of neocortical cells, but are also deeply involved in cognitive functions and related disorders [68,153]. Therefore, it is not surprising that experimental models of neuroinflammation characterized by cognitive impairment display various structural and functional alterations in PFC microcircuits elements, including PNs and INs, with consequences that ultimately converge on E/I balance.

For instance, using in vivo recordings, Yang et al. [131] show that delay-related sustained firing in putative PNs of the DLPFC of aged macaque monkeys is reduced after iontophoretic application of kynurenic acid. Unfortunately, owing to the great variety of experimental procedures used to induce inflammatory reactions in the CNS, and to the different methods adopted for the study of PN functional properties, the scientific literature in this regard is far from a consensus.

Using whole-cell patch clamp recordings of L2/3 PNs in the PFC of mice 24 h after systemic administration of LPS, Diaz-Castro et al. [149] found no alterations in sub- and suprathreshold parameters, nor in excitatory postsynaptic currents (EPSCs).

Using a similar protocol based on LPS injection, a reduction in the ratio between inhibitory postsynaptic currents (IPSCs) and EPSCs has been observed in L5 PNs of the mPFC of mice at earlier time points after treatment (8 h) [154]. In the same study, a decreased amplitude of IPSCs was found in L5 PNs after bath application of the proinflammatory cytokine interleukin 6 (IL-6) to slices of mPFC [154]. In PNs of L2/3 and L5 of the mPFC, the application of IL-1β enhances the intrinsic excitability, in terms of action potential (AP) threshold and firing frequency, whereas only a slight increase in excitability was observed after intraperitoneal LPS injection [155]. The changes in intrinsic excitability can be explained by mechanisms which, though not completely elucidated, may include the direct interaction of inflammatory mediators, such as IL-1β, with voltage or ligand-gated ion channels (see below and see [155,156] for a discussion on this point). From the works cited above, it appears that the LPS model of neuroinflammation is characterized by early occurring hyperexcitability of PNs. In disagreement with this view, Jiang et al. [157] demonstrate an increase in IPSCs in PNs of L2/3 PL cortex 2 h after LPS exposure. Decreased excitability of L5 PNs of the IL cortex was observed using a model of repeated administration of LPS [158]. Another recent study carried out with repeated administration of LPS (through 7 consecutive days) demonstrated a shift in the E/I balance to inhibition in PNs of the mPFC, using both ex vivo patch clamp recordings and in vivo Ca^2+^ imaging [159]. The different protocols of LPS exposure (single vs. repeated injections) and different timeframes of observation can account for the contradictory results obtained in these studies.

In addition to the findings obtained in the widely used LPS model, changes in excitability of PNs have also been shown in other models of neuroinflammation. Increased excitability of PL L5 PNs has been found in whole-cell current clamp recordings after injection of Freund’s adjuvant [160]. Using the same model, Wei et al. [161] show that astrocytic activation is accompanied by a shift in E/I balance to excitation in L5 PNs of the ACC. Papadogiannis and Dimitrov [162] explain the hyperexcitability observed in the CFA model, showing an increase in dendritic spine density in the mPFC PNs of male mice. Conversely, a decreased density of spines and reduced dendritic branching was observed in L2/3 PNs of mPFC after inflammation induced by HIV proteins [163]. Interestingly, these authors found a significant effect only on basal (Figure 1) and not on apical dendrites. Hyperexcitability of L5 PL PNs has also been shown after toluene exposure and is likely mediated by inflammation [164].

Although neuroinflammation may affect the expression and function of ion channels, there are only a few findings regarding HCN channels in the PFC. Indeed, the inhibition of HCN1 channels by type 1 interferons (IFNs) has been demonstrated in PNs of the somatosensory cortex [165]. Likewise, a 50% decrease in the Ih current was observed in CA1 hippocampal neurons after ICV injection of LPS [166]. However, there is only one study investigating Ih currents in PFC PNs, although during low grade neuroinflammation induced by high-fat diet. In particular, Feng et al. [167] show enhanced excitability of L2/3 PNs of the PL cortex, along with an increase in the Ih current. It should be noted that, despite conflicting results in different cortical areas, due to the complex effect of the Ih current on neuronal excitability, both the up- and downregulation of HCN channels can lead to functional impairment of PNs [90]. As for ligand-gated ion channels involved in glutamatergic transmission, Zubareva and coworkers found no change in NMDA and AMPA receptor subunits in the PFC of young rats exposed to LPS [168]. By contrast, a downregulation of the synaptic GluR1 AMPA receptor has been found in adult mice treated with LPS [169].

Finally, it should be pointed out that disorders of cortical development that transiently or permanently impact the structure and function of PNs are at least partly mediated by neuroinflammation. Prenatal exposure to LPS leads to reduced dendritic length in L2/3 PNs of the mPFC during the first stage of postnatal life, with a return to normal values at adulthood, while the reduction in spine density in L5 neurons is permanent [170]. The cytoarchitectonics and the functional properties of the PFC can thus be impaired in neurodevelopmental disorders characterized by inflammatory activation, such as fetal alcohol spectrum disorders [171,172].

All these changes are schematically represented in Table 1.

### 4.2. Interneurons and Inflammation

It is known that cytokines, such as IL-1β [173], TNF-α [174,175], IL-6 [176], IFN-γ [177], and other mediators of inflammation affect GABAergic transmission, leading to disruption of the E/I balance, crucial for the proper function of cortical microcircuitry. In particular, E/I imbalance in PFC has been shown to underlie cognitive impairment and neuropsychiatric diseases, including schizophrenia, autism, and intellectual disabilities [178].

In the PFC, neuroinflammation affects GABAergic transmission [154,159], inducing changes in the integrity and function of specific interneuron subpopulations.

Growing evidence highlights the relevance of dysfunctional changes affecting the highly vulnerable PV+ INs. In fact, through their fast-spiking inhibitory activity, they modulate the glutamatergic activity of PNs, thereby controlling specific functional properties of PFC, such as high-order cognitive functions and complex behaviors [179,180,181,182]. Changes affecting this IN subpopulation have been widely described in different psychiatric and neurodegenerative diseases [27,183], all of which are associated with local neuroinflammation. Their specific vulnerability to neuroinflammatory insults has been related to their fast-spiking activity, which requires a high metabolic rate, making them susceptible to many stressors [27,184,185]. In this regard, numerous findings support the notion that acute neuroinflammation induced by systemic LPS administration severely affects the density and functional properties of these cells in the PFC. In the rat mPFC, for instance, microglial activation following repeated systemic LPS doses reduces GABAergic markers, such as Glutamate Decarboxylase 67 kDa (GAD67) and Vesicular GABA Transporter (vGAT), as well as PV expression, thereby altering neural oscillations [186]. Moreover, the PFC of mice exposed to a single dose of LPS [187] or LPS combined with chronic unpredictable stress [188] displays decreased PV expression and disturbances in theta and gamma oscillations, associated with cognitive impairment [188]. However, in addition to these observations suggesting reduced PV+ IN activity as a response to acute neuroinflammatory insult, opposite findings have also been reported (Figure 1 and Table 1). For instance, in the single-dose LPS model, increased intrinsic excitability, selectively involving PV+ INs in the PL mPFC, was observed 6 h after treatment, without changes in PV+ cell numbers or *Pva* gene mRNA expression levels [189].

These findings align with those in mice euthanized 2 h after LPS administration, which point to upregulation of GABA signaling proteins, such as GABA_A_ R α1, β2, α5 and γ2 subunits, glutamine synthetase, GAD67/65, and vGAT [157], suggesting an increase in inhibitory activity, leading to the upregulation of mIPSCs in mPFC PNs. Moreover, in the mPFC of mice treated with multiple LPS doses, a late-onset increase in GABAergic markers such as _V_GAT, together with increased numbers of both PV+ cells and PV+ boutons on PNs, was observed, in association with increased inhibitory activity on PNs [159]. Although sustained by a different pathogenetic mechanism of neuroinflammation, the latter results are in line with those found in the experimental autoimmune encephalomyelitis (EAE) model of Multiple Sclerosis performed in SJL/J mice, an example of a primary inflammatory disease of the CNS, in which inflammation-dependent increased numbers of PV+ INs and increased *Pva* mRNA expression levels were detected in the mPFC at the peak of the acute phase of the disease [190].

In another model of autoimmune CNS inflammatory disease, the anti-NMDAR encephalitis, a significant reduction in the dendritic tree complexity of mPFC PV+ neurons, reduced excitability, synaptic dysfunction and loss of gamma oscillations induced by NMDA antagonists were reported [191]. Notably, activation of mPFC PV+ INs reversed the cognitive impairment induced by anti-NMDAR IgG. At the molecular level, single-cell analysis of PV+ INs revealed downregulation of genes associated with synaptic plasticity and neuronal development [191].

The reported discrepancies may be due to several reasons, including differences in experimental animal species (rat vs. mouse) or strains (C57Bl/6J vs. SJL/J), in experimental models and/or protocols of neuroinflammation (single-dose LPS, repeated-dose LPS, EAE), the time frame of investigation (early or late time points after the inflammatory insult), and the methods of quantitative analysis used in the studies (e.g., fluorescence intensity, cell density, cell counts performed with semiquantitative vs. stereological methods). Overall, the reported findings highlight the important notion that fluctuations in PV expression observed in different experimental conditions indicate functional changes in these INs, more than their neurodegeneration or loss [192,193,194].

SST+ INs are another subpopulation of interneurons extensively studied in pathological conditions [195]. They are particularly abundant in the PFC (see Section 2.3.2), where they have been divided into SST-Martinotti cells, which contact the dendritic tree of PNs, and non-Martinotti cells, contacting PV+ INs, thus producing disinhibition of PNs [71]. Notably, even though the disinhibitory circuit motif throughout the neocortex is mediated by VIP+ INs (see Section 2.3.1), PFC disinhibition might involve SST+ INs primarily (Figure 1). Indeed, top-down inputs are the main source of activation of VIP+ INs in the cortex, but the source of top-down projections in the PFC is a matter of debate, due to the high position of this area in cortical hierarchy [196]. This may represent an essential mechanism in the regulation of prefrontal functions, such as learning and social fear conditioning (for review see [197,198]).

Also in this case, there is evidence supporting the involvement of SST in the changes in PFC microcircuits specifically induced by neuroinflammation, although the results are not homogeneous. Twenty-four hours and 18 h after single-dose LPS administration, a downregulation of the *Sst* gene, as well as of those encoding other neuropeptides frequently expressed by SST+ INs, such as neuropeptide y (*Npy*) and cortistatin (*Cort*), has been detected in the PFC of treated mice [149,187]. Notably, the same result emerges from a genome-wide transcriptomic analysis of the mPFC of SJL/J EAE mice analyzed at the peak of the acute phase of the disease [199], although the inflammatory process affecting the PFC differs in the two models (peripherally induced vs. autoimmune-induced neuroinflammation). Inflammation-induced decrease of brain derived neurotrophic factor (BDNF) expression has been proposed as a possible mediator of the observed reduction in SST+ IN markers [187,199], due to the high sensitivity of these cells to the BDNF-Trkb signaling [200].

However, also in this case, conflicting results are present. Indeed, in both rats and mice treated with repeated systemic doses of LPS, no changes in PFC SST+ IN subpopulation were observed at later time points after treatment [159,186]. It may be hypothesized that the reported variations in SST expression could reflect plastic and possibly transient functional states of PFC network activity in different phases of the inflammatory process.

There are only a few observations regarding VIP+ INs during neuroinflammatory processes of the PFC. In particular, Rezaei and coworkers did not observe variations in the expression of markers of this interneuron subpopulation after LPS injection [187], thus supporting the hypothesis that the role of VIP INs in the PFC microcircuitry might be slightly different compared to other cortical areas (see above).

It is known that early-life inflammation leads to neurodevelopmental pathologies [201], and PV+ INs, which are characterized by late postnatal development [202], appear particularly sensitive to early-life insults [203,204]. In this regard, selective vulnerability of PV+ INs has been found in several models of early-life insults associated with neuroinflammation [205], including maternal immune activation [206], inflammation associated with early-life stress [207,208], and early-life immune activation induced by perinatal LPS administration [209]. Also in this case, opposite results have been reported in a rat model of maternal immune activation obtained with LPS administration, in which prenatal LPS treatment significantly increased the density of PV- and CCK-immunoreactive neurons in the mPFC and DLPFC, as well as in the ventral subiculum of offspring at P14, while the reduction is limited to SST+ INs [210]. Conversely, an increase in SST+ cell number in the ACC has been reported in female mice, but not in males, after neonatal LPS challenge, resulting in a long-lasting impairment of social behaviors [211]. At the network level, neuroinflammation may initially induce compensatory changes in inhibition or excitation, depending on the timing and intensity of the insult. All these changes are schematically represented in Table 1.

Taken together, these findings suggest that PFC inflammation leads to E/I imbalance, which might represent the structural basis of the cognitive impairment associated with neurodevelopmental pathologies (Table 1).

**Table 1:** Prefrontal cortex Microcircuit alterations induced by neuroinflammation. Table 1 summarizes the main experimental studies, stratified according to inflammatory model, temporal profile (acute vs. chronic), developmental stage, and main circuit-level findings. Abbreviations. M/F: male and female; dpt: days post treatment; PNs: Pyramidal Neurons; LPS: lipopolysaccharide; EPSCs: excitatory postsynaptic currents; Sstr: SST receptors; *Cck:* Cholecystokinin; i.p.: intraperitoneal; s.c.: subcutaneously ICR: Institute of Cancer Research; CFA: complete Freund’s adjuvant; tg: transgenic; CRH: corticotropin-releasing hormone; CUS: Chronic unpredictable stress; HIV(Tg): Hsd:HIV-1(F344) transgenic rats; MyD88-floxed (F/F) and either Cre-: no modification to microglial MyD88; Cre+ (Cre tg/0): removal of microglial MyD88; ACC: Anterior Cingulate Cortices; ppm: parts per million; ↓: decrease/reduction; ↑: increase; ↔: no changes.

Models of Neuroinflammation	Species/Strains	Sex	Age	Acute/Chronic	Timeframes	Main Results	Ref.
Model of aging	Macaque monkey	M/F	24–31 yo	Chronic	24–31 years	↓ delay-related sustained firing in PNs.	[131]
LPS administration (5 mg/kg, i.p., single dose)	C57BL/6J mice	M	Adult	Acute	2 dpt: PN registration and 1 dpt: RNA-seq analysis.	↔ sub-/suprathreshold parameters, ↔ EPSCs in L2/L3 PNs. ↓ *Sst*, *Sstr2*, *Sstr3*, *Cck.*	[149]
LPS administration (10 mg/kg, i.p., single dose)	C57BL/6J Sprague Dawley rats	N/A	Adult	Acute	8 h after LPS administration	↓ amplitude of IPSCs in PNs.	[154]
LPS administration (2 mg/kg, i.p., single dose)	C57BL/6N mice	N/A	Adult	Acute	4, 6, 8, or 24 h after LPS administration	PNs of L2/3 and L5: slight ↑ excitability. ↓ δ power 2–8 h; ↓ θ and ↓ γ power 4–8 h; ↓ high γ power at 8 h. Altered fronto-occipital functional connectivity.	[155]
LPS administration (0.5 mg/kg, i.p., single dose)	C57BL/6J mice	M/F	Adolescent/Adult	Acute	2 h after LPS administration	↑ mIPSCs in PNs of L2/3 PL cortex. ↑ GABA signaling; ↑ GABA content/release	[157]
LPS administration 2 mg/kg/day i.p. for 3 days	ICR mice	M	Adult	Acute	24 h after the last LPS injection	↓ excitability of L5 PNs	[158]
LPS administration 0.5 mg/kg/day i.p. for 7 days	C57BL/6J mice and PV-Cre mice	M	Adult	Chronic	24 h after behavioral tests (11 days after treatment)	↑ PV+ cells; ↑ _V_GAT+ and ↑ PV+ boutons on PNs; ↑ inhibition of PNs. ↔ SST INs.	[159]
Intraplantar administration of CFA (peripheral inflammation)	ICR mice	M	Adult	Acute	3–5 days after CFA/saline injection	↑ excitability of PL L5 PNs	[160]
Intraplantar administration of CFA (peripheral inflammation)	Outbred CD-1 mice	M/F	Adult	Acute	7 days after treatment	↑ total spinal density of PNs (only male), and of INs (M/F)	[162]
HIV-induced neurocognitive disorders/neuroinflammation	HIV(Tg) rats and controls	N/A	Adult	N/A	N/A	↓ spine density in apical and basal dendrites L2/3 PNs	[163]
Toluene exposure (8000 ppm/30 min/twice a day, 6 h apart, for 5 dd; untreated for 2 dd and re-exposed for 5 dd)	Wistar rats	N/A	Adolescent	Acute	24 h after last exposure	↑ intrinsic excitability and firing frequency of mPFC L5 PL PNs.	[164]
High-fat diet for 18 weeks starting from 5 weeks of age	C57BL/6J mice	M/F	Adult	Chronic	After diet and behavioral tests	↑ excitability and ↑ Ih currents in L2/3 PNs of the PL (male)	[167]
Prenatal immune challenge (LPS 100 μg/kg i.p./ E15/E16)	Sprague-Dawley rats	F	Adult	Chronic	P10, P35 and P60	↓ total dendritic length of L3 PNs at P10 and P35, ↔ P60.	[170]
Sepsis induced by cecal ligation and puncture (CLP)	C57BL/6 mice	M	3–4 mo	Chronic	14 and 30 days after CLP	↓ expression of PV in PFC. ↔ in SST INs.	[186]
LPS administration (2 mg/kg i.p. single dose)	C57BL/6 J mice	M/F	8 weeks	Acute	18 h after LPS administration	↓ *Sst*, *Pv*a, *Npy* and *Cck.* ↔ *CRH* and *Vip*.	[187]
LPS administration (3 mg/kg i.p. single dose) and CUS	C57BL/6 mice	M	3–4 mo	Chronic	About 30 days after LPS+CUS treatment	↓ intensity of PV immunostaining. ↓ θ/γ oscillations.	[188]
LPS administration (0.3 mg/kg i.p. single dose)	C57BL/6J mice	M	8–10 weeks	Acute	6 h following LPS administration	↑ excitability of PV INs (↑ input resistance, ↓ rheobase current, ↑ frequency action potentials).	[189]
EAE	SJL/J mice	F	6–8 weeks	Acute	post immunization day 16 ± 2	↑ numbers of PV+ INs ↑ *Pv*a, ↓ *Sst*	[190,212]
Model of Anti-NMDAR autoimmune encephalitis (Stereotactic injection of patient’s derived Anti-NMDAR antibodies)	C57BL/6J mice; PV-Cre mice.	M	6–8 weeks 4–8 weeks	Acute	3 days after treatment	↓ dendritic tree complexity in PV INs. ↓ PV INs excitability; ↓ amplitude/frequency sEPSCs in PV Ins; ↓ IPSC frequency with ↔ amplitude in PNs; ↓ γ oscillations. ↓ synaptic plasticity and neuronal development associated genes.	[191]
LPS administration at gestation days 15 and 16	Rats	N/A	N/A	Chronic	P14	↑ density of PV and CCK INs; Reduction in SST Ins.	[210]
Neonatal immune challenge (LPS 330 μg/kg s.c. at P4)	MyD88-floxed/Cre^0/0^ or Cre^tg/0^ mice;	N/A	N/A	Chronic	P60	↑ SST IN number in the ACC.	[211]

Discrepancies may be the consequence of differences in species, sex, inflammatory paradigms, timing of assessment, and experimental conditions. Divergent results across models might also be related to the intensity and duration of the inflammatory stimulus that may critically influence neuronal outcomes. This is further supported by a recent study, which provides molecular evidence of IN marker downregulation only in mice with high levels of PFC inflammation [212] (Table 1). Prolonged or severe inflammatory conditions are more likely to result in unstable E/I balance, impairing PFC-dependent computations such as working memory and cognitive flexibility. Although there are not many studies examining, at the network level, the consequences of neuroinflammatory events in the PFC, the occurrence of changes in intrahemispheric connectivity emerges. In particular, in adult rats treated with a single dose of LPS, a transient slowing of the electroencephalogram (EEG) was observed 6 h following LPS administration, associated with increased slow wave power (delta and theta bands) and reduced gamma/high frequency oscillations [213]. The authors also found changes in both intra- and interhemispheric functional connectivity [213]. Although of opposite sign, in LPS-treated mice, Mittli et al. observed similar changes, represented by a general reduction in delta, theta, and gamma oscillations measured by epidural EEG recordings of the frontal cortex after exposure to LPS, along with a significant increase in fronto-occipital functional connectivity [155]. Due to the relevance of these connections in working memory and the top-down regulation of visual processing [43], these findings add elements that are useful for understanding the connection between neuroinflammation and impairment of cognitive functions.

## 5. Conclusions

It has been widely described that neuroinflammation underlies cognitive impairment; especially when the inflammatory process involves brain regions regulating emotional behaviors and high-level cognitive processes, such as the PFC. Indeed, prolonged postnatal development, specialized microcircuit organization and region-specific properties of resident immune cells make the PFC particularly susceptible to neuroinflammatory events. In this review, we have summarized recent findings emerging from preclinical studies in different experimental models of neuroinflammation involving the PFC, dissecting how inflammatory processes impair the anatomical and functional features of PFC PNs and INs. Although conflicting results emerge from our analysis, the main message is that inflammatory processes involving the PFC induce E/I imbalance, based on the rewiring and possibly the inflammation-induced instability of the local microcircuits. However, the reported findings could also be the result of compensatory processes aimed at restoring local homeostasis after the inflammatory insult, reflecting adaptive local responses that preserve or restore impaired functions. It is known that homeostatic responses can become detrimental ones when prolonged [214]. In this regard, a more accurate exploration of the long-term effects of inflammatory insults, still poorly explored, will be useful to clarify how neuroinflammation reshapes PFC microcircuits.

A major challenge in understanding how neuroinflammation affects the brain, and in particular the PFC, lies in explaining how molecular events induced by inflammatory mediators give rise to changes in neuronal dynamics and, ultimately, in cognition.

Future studies, integrating accurate spatio-temporal resolution of inflammation-induced events, longitudinal designs, high-resolution circuit analysis, behavioral readouts and circuit-level functional analyses, will be essential to unequivocally dissect PFC responses to neuroinflammation. In a translational perspective, computational studies focused on neuroinflammation-induced changes in neuronal dynamics of PFC microcircuitry might provide insights useful to bridge the macroscale levels to microscale events, provided by mechanistic studies about the molecular events underlying neuronal dysfunction. They may represent an integrated framework able to capture feedback between neuronal dynamics and disease biology, indispensable to identify therapeutic strategies aimed at preserving neuronal integrity in inflammatory brain disorders [215].

Future studies should therefore (i) prioritize longitudinal designs, (ii) cell-type-specific approaches (pyramidal vs. interneurons), (iii) analyses of sex differences, and (iv) the integration of vascular, glial, and synaptic readouts. A more mechanistic understanding of how inflammatory burden, timing, and duration shape PFC circuit responses will be essential not only to reconcile apparently conflicting findings across models but also to identify testable therapeutic strategies aimed at preserving cognitive function in inflammatory brain disorders.

## 6. Limitations and Methodology

This review has several limitations. First, it is a narrative and non-systematic review and therefore does not utilize a formal quality assessment framework. Second, most of the evidence discussed derives from rodent preclinical models, which provide mechanistic insight but only partially capture the complexity of human neuropsychiatric and neuroinflammatory disorders. Third, marked heterogeneity across inflammatory paradigms, species, sex distribution, developmental stage, and electrophysiological or morphological parameters limits direct comparability across studies. Finally, widely used models, such as systemic LPS administration, are experimentally valuable for probing inflammatory mechanisms, but they should not be interpreted as direct models of specific human neurologic or psychiatric disorders, of which it reproduces only some features [11]. Moreover, most studies are carried out on rodents, whose prefrontal fields are not comparable to those of primates [35].

Given the narrative nature of this article, studies were selected based on conceptual relevance to PFC microcircuit dysfunction under inflammatory conditions. Literature searches were conducted in PubMed and Scopus using combinations of terms such as “prefrontal cortex”, “neuroinflammation”, “encephalitis”, “LPS”, “CFA”, “EAE”, “microglia”, “astrocytes”, “interneurons”, “pyramidal neurons”, and “cytokines”. Priority was given to primary experimental studies, whereas selected review articles were used for conceptual framing.

Since the term “neuroinflammation” is used to describe pathological features of a very heterogeneous and large group of diseases [216], we focused specifically on experimental and preclinical models of neuroinflammation, in which inflammatory processes primarily involve the CNS, such as EAE [190] and different forms of encephalitis (CFA administration, anti-NMDA encephalitis). Due to its effectiveness in mimicking neuroinflammation and to its wide use, we included models based on systemic administration of LPS, used to study not only neuroinflammatory processes associated with peripheral challenge (for review see [11]), but also those associated with psychiatric [217], and neurodegenerative diseases [36]. Models in which inflammation is low and represents an epiphenomenon of the whole pathologic picture (e.g., models of stress, Alzheimer’s disease, Parkinson’s disease) were not included, unless they provide a significant conceptual contribution to the understanding of specific changes that PFC microcircuitry undergoes during neuroinflammation [167].

Notably, most studies are not sex-balanced, limiting conclusions about sex-specific effects of neuroinflammation on PFC circuits.

## Data Availability

Not applicable.

## Abbreviations

The following abbreviations are used in this manuscript:

PFC	Prefrontal Cortex
CNS	Central Nervous System
AD	Alzheimer’s Disease
TNF-α	Tumor Necrosis Factor-alpha
ROS	Reactive Oxygen Species
LPS	LipoPolySaccharide
L4	layer 4
MD	MedioDorsal
OFC	OrbitoFrontal cortex
vmPFC	VentroMedial PreFrontal Cortex
DLPFC	DorsoLateral PreFrontal Cortex
VLPFC	VentroLateral PreFrontal Cortex
DMPFC	DorsoMedial PreFrontal Cortex
IL	InfraLimbic
PL	Pre-Limbic
ACC	Anterior Cingulate Cortices
PNs	Pyramidal Neurons
L2/3	supragranular layers
INs	Inhibitory GABAergic interneurons
PV	Parvalbumin
SST	Somatostatin
VIP	Vasoactive intestinal peptide
Ih	hyperpolarization-activated current
sADP	slow afterdepolarization
HCN	Hyperpolarization-activated Cyclic Nucleotide-gated
VGCCs	voltage-gated Ca^2+^ channels
NMDA	N-methyl-D-aspartate
D1/D2	Dopamine receptors
nNOS	Neuronal Nitric Oxide Synthase
ChAT	choline acetyltransferase
CFA	Complete Freund’s adjuvant
CA1	*Cornu Ammonis* 1
IFN	Interferon
EAE	Experimental Autoimmune Encephalomyelitis
GAD67	Glutamate Decarboxylase 67 kDa
vGAT	Vesicular GABA Transporter
NPY	Neuropeptide Y
LTP	Long-Term Potentiation
E/I	Excitation/Inhibition
CCK	Cholecystokinin
EPSCs	Excitatory PostSynaptic Currents
5HT3aR	Serotonin Receptor 3a
IL-6	InterLeukin 6
IL-1β	InterLeukin 1β
IPSCs	Inhibitory Postsynaptic Currents
